# Narrative Review of Body-First Versus Brain-First Prodromal Neuropsychiatric Symptoms in Parkinson’s Disease

**DOI:** 10.7759/cureus.110371

**Published:** 2026-06-06

**Authors:** Saacha F Mohammed, Anshika Goyal, Shradha P Kakde, Anushka P Mishra, Ashik Kumar Ashok Kumar, Doreen Chepng'eno Koske, Bashir Imam, Jeby Abraham, Juviel Rev Reyes Cruz, Aaron Mathew Jacob, Somya Majji, Shruthi Aswathappa

**Affiliations:** 1 Medicine, Trinity College, University of Dublin, Dublin, IRL; 2 Faculty of Medicine, Maulana Azad Medical College, New Delhi, IND; 3 Medicine and Surgery, Mahatma Gandhi Mission Institute of Health Sciences, Navi Mumbai, IND; 4 Medicine, Era's Lucknow Medical College and Hospital, Lucknow, IND; 5 Faculty of Medicine, UV Gullas College of Medicine, Cebu City, PHL; 6 Faculty of Medicine, University of Nairobi, Nairobi, KEN; 7 Pediatrics, University of Pittsburgh Medical Center, Pittsburgh, USA; 8 Faculty of Medicine, Yenepoya Medical College, Mangalore, IND; 9 Faculty of Medicine, University of the East Ramon Magsaysay Memorial Medical Center, Quezon City, PHL; 10 Faculty of Medicine, Alpharetta High School, Alpharetta, USA; 11 Faculty of Medicine, Mahadevappa Rampure Medical College, Kalaburagi, IND; 12 Medicine and Surgery, M.S. Ramaiah Medical College, Bengaluru, IND

**Keywords:** alpha synuclein, anxiety, biomarkers, depression, mild cognitive impairment, parkinson disease, prodromal symptoms, rem sleep behavior disorder

## Abstract

Parkinson’s disease (PD) is a multisystem neurodegenerative disorder in which nonmotor symptoms often precede classical motor manifestations by years. Neuropsychiatric symptoms such as REM sleep behavior disorder (RBD), depression, anxiety, apathy, and mild cognitive impairment are increasingly recognized as clinically meaningful prodromal features, yet they remain underrecognized in routine practice. This narrative review summarizes literature identified through PubMed, Scopus, and Google Scholar between 2003 and 2025 using terms related to PD, prodromal symptoms, neuropsychiatric symptoms, alpha-synuclein, and body-first versus brain-first disease. Priority was given to review articles, original studies, clinical trials, Movement Disorder Society prodromal criteria, and foundational neuropathological models. Current evidence supports a dual pathway model of PD progression. In the body-first phenotype, pathology may begin in the peripheral autonomic or enteric nervous system and ascend through the brainstem, where RBD and autonomic dysfunction are prominent early markers. In the brain-first phenotype, pathology is proposed to arise in olfactory or limbic regions, where hyposmia, anxiety, and depression may precede motor disease. Prodromal neuropsychiatric symptoms are among the earliest clinically detectable features of PD. Interpreting these symptoms within the body first versus brain first framework may improve risk stratification, earlier recognition, and selection of candidates for biomarker-driven and disease-modifying studies.

## Introduction and background

Parkinson’s disease (PD) is a progressive multisystem neurodegenerative disorder in which nonmotor symptoms frequently precede the onset of classical motor features by several years [1]. Among the most clinically relevant prodromal manifestations are neuropsychiatric symptoms, including depression, anxiety, apathy, sleep disturbances, early cognitive change, and distress associated with olfactory dysfunction [2-3]. These symptoms are being interpreted within a dual pathway model of alpha synucleinopathy, in which disease may begin predominantly along a body-first route or a brain-first route [4-8].

**Figure 1:**
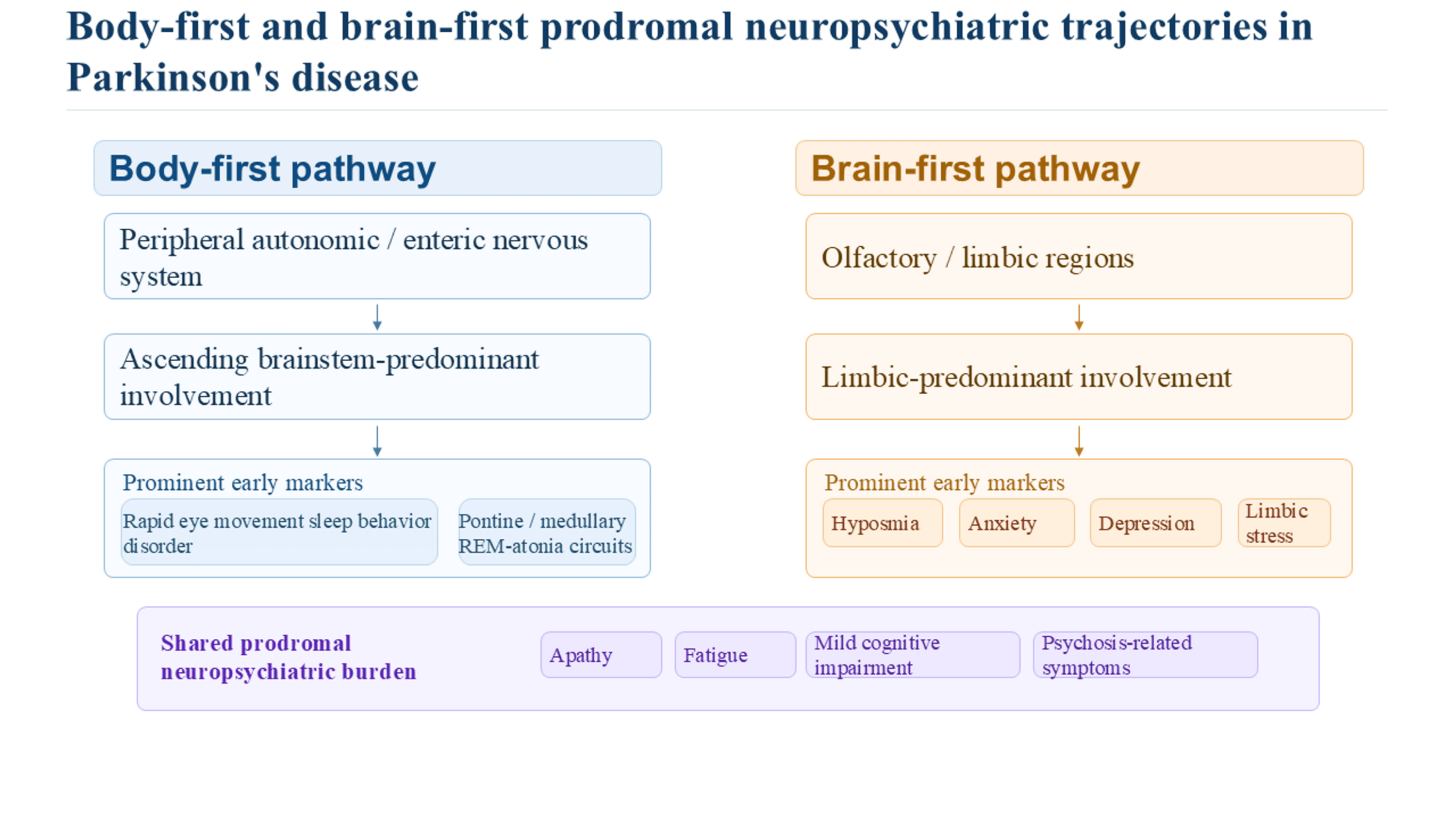
Conceptual schematic of body-first and brain-first prodromal neuropsychiatric trajectories in Parkinson’s disease. Image credit: Saacha Mohammed. This figure was created using Microsoft PowerPoint.

In the body-first model, pathology is proposed to begin in the peripheral autonomic or enteric nervous system and then ascend through the brainstem, with REM sleep behavior disorder (RBD) and autonomic dysfunction emerging early. In the brain first model, pathology may begin in olfactory or limbic structures, where hyposmia, anxiety, and depression may appear before significant brainstem involvement [4-8]. Because these prodromal features reflect dysfunction across dopaminergic, serotonergic, noradrenergic, and cholinergic networks, they may provide a window for earlier recognition and risk stratification in high-risk groups [9-12]. Figure 1 illustrates the pathways.

In the body-first pathway, pathology is proposed to begin in the peripheral autonomic or enteric nervous system and ascend through brainstem circuits, with rapid eye movement sleep behavior disorder and autonomic dysfunction as prominent early markers. In the brain-first pathway, pathology is proposed to arise in olfactory or limbic regions, where hyposmia, anxiety, and depression may precede major brainstem involvement. Both pathways may later converge into a broader prodromal neuropsychiatric burden characterized by apathy, fatigue, mild cognitive impairment, and psychosis-related symptoms; clustering of markers may improve near-term risk stratification for Parkinson’s disease [4-9,12-47].

## Review

Methodology

This narrative review was based on a literature search of PubMed, Scopus, and Google Scholar for articles published between 2003 and 2025. Search terms included PD, prodromal symptoms, neuropsychiatric symptoms, alpha-synuclein, and brain-first versus body-first. We prioritized review articles, observational studies, longitudinal cohorts, clinical trials, MDS research criteria, and foundational neuropathological models addressing neuropsychiatric manifestations before the onset of motor PD. Because this is a narrative rather than a systematic review, studies were synthesized qualitatively to provide an integrative overview of the pathophysiology, clinical relevance, and future diagnostic implications of prodromal neuropsychiatric symptoms.

Understanding the prodromal phase of PD

The prodromal phase of PD is characterized by non-classical symptoms that precede diagnostic motor manifestations, indicating that neurodegenerative processes are already underway [4-8]. Some authors and colleagues have proposed that PD-related pathology may begin in the enteric nervous system and olfactory bulb before spreading to the brainstem, substantia nigra pars compacta (SNc), and cortex [4]. Although this staging model does not account for every clinicopathological pattern, it remains highly influential in conceptualizing early disease spread [5-8,48].

Alpha-synuclein aggregation impairs mitochondrial function, disrupts synaptic signaling, and contributes to Lewy pathology [4-8,48]. Importantly, alpha-synuclein-related changes in serotonergic neurons of the raphe nuclei and noradrenergic neurons of the locus coeruleus (LC) may occur before substantial nigrostriatal dopaminergic loss, corresponding to early symptoms such as depression, anxiety, apathy, and RBD [49-51]. Imaging studies using neuromelanin-sensitive magnetic resonance imaging (MRI) and positron emission tomography (PET) further support early LC involvement in prodromal states [10-11].

Body-first phenotype: brainstem predominant markers

RBD is a parasomnia characterized by dream-enactment behaviors caused by loss of physiologic REM atonia and is often accompanied by vocalization or complex motor activity during sleep [18-19]. Idiopathic RBD is one of the strongest clinical predictors of an underlying alpha-synucleinopathy, especially PD and dementia with Lewy bodies [9,13,18-19]. According to MDS prodromal criteria, RBD carries the highest likelihood ratio among established prodromal markers, underscoring its major clinical weight in risk estimation [9,13]. Longitudinal data summarized in recent reviews suggest that a substantial proportion of individuals with idiopathic RBD convert to PD or a related synucleinopathy over follow-up, including rates approaching 73% within 12 years in some cohorts [19].

Neuroimaging findings in idiopathic RBD are consistent with early nigrostriatal involvement. Studies show reduced striatal transporter binding compared with healthy controls, although deficits are often less severe than in established PD [52-54]. PET studies using vesicular monoamine transporter 2 (VMAT2) ligands and related tracers also support presynaptic dopaminergic terminal loss in this population [55]. In parallel, alpha-synuclein has been detected in cerebrospinal fluid, skin, and other peripheral tissues in selected prodromal cohorts, reinforcing the concept of idiopathic RBD as an early synucleinopathy rather than an isolated sleep disorder [18-19].

Experimental models further support early dysfunction within REM atonia circuits of the pontine tegmentum and medulla, where alpha-synuclein deposition can disrupt glutamatergic and GABAergic pathways involved in motor inhibition during REM sleep [48,56]. Clinically, autonomic symptoms such as constipation often cluster with RBD and may emerge years before motor onset, a pattern that is particularly compatible with the body-first trajectory [8,12,56].

Brain-first phenotype: limbic and olfactory markers

Anxiety is a common non-motor symptom in PD and may affect roughly one-third of patients [20]. In a prodromal context, it may reflect early dysfunction within limbic and paralimbic networks rather than a purely coincidental psychiatric disorder [21-24]. Neuroimaging studies have implicated the amygdala, anterior cingulate cortex, hippocampus, and limbic cortico-striato-thalamo-cortical circuits, with evidence of altered connectivity and regional structural change in patients with PD-related anxiety [21-24].

Depression is also common in PD and may precede motor diagnosis in a subset of patients [25-26]. Its neurobiology appears multifactorial, involving serotonergic, noradrenergic, dopaminergic, inflammatory, and network-level mechanisms [25-26,57-59]. Transcriptomic and single-cell analyses of postmortem tissue suggest that PD-associated depressive syndromes may involve neuroinflammatory and oligodendroglial changes that are not fully explained by primary major depressive disorder, although these findings should be interpreted cautiously and not as definitive proof of prodromal PD in every patient with depression [57-59].

Olfactory dysfunction is another hallmark of the brain-first hypothesis and may precede motor symptoms by many years [14]. Hyposmia or anosmia is reported in most patients with established PD and alpha-synuclein pathology in the olfactory bulb [4,14,60]. Because olfactory dysfunction is common and non-specific, it is most informative when interpreted alongside other prodromal markers rather than in isolation [9,13].

Convergent symptoms: broader neuropsychiatric involvement

Apathy is a subtle but clinically important prodromal feature characterized by reduced motivation, diminished goal-directed behavior, and emotional blunting [27-29]. It is distinct from depression, although overlap is common, and has been linked to dysfunction of prefrontal-basal ganglia circuits, particularly the anterior cingulate cortex and medial orbitofrontal cortex [15,29-30]. Imaging studies demonstrate reduced dopaminergic signaling and abnormal reward processing in apathetic PD populations, supporting the view that apathy may mark early network degeneration rather than simply a reactive mood change [31-36].

Mild cognitive impairment (MCI) represents a critical intermediate stage between prodromal dysfunction and PD dementia [37-39]. In early PD, MCI commonly manifests as executive and attentional impairment and has been associated with dopaminergic depletion, cholinergic dysfunction, hippocampal vulnerability, and cortical Lewy pathology [38-44,51,61]. Fatigue and psychosis-related phenomena may also emerge along the prodromal spectrum, although their specificity is lower than that of RBD [16-17,45]. Impulse control disorders are often medication-related in established PD and should not be over-interpreted as core untreated prodromal markers [62].

Recent population-scale data also suggest that the cumulative burden of prodromal features matters. Clustering multiple prodromal PD features is associated with a higher short-term risk of incident PD, supporting the concept that multisystem involvement may mark a transition from isolated symptoms to clinically meaningful neurodegenerative risk [46-47]. Table 1 lists all prodromal symptoms.

**Table 1:** Summary of neuropsychiatric prodromal markers of Parkinson's disease. Estimated onset refers to the approximate interval in years before the onset of diagnostic motor Parkinson’s disease. Prevalence and likelihood-ratio ranges are approximate and should be interpreted within a multivariable prodromal framework rather than as stand-alone diagnostic measures. Phenotype assignment reflects the dominant proposed trajectory in the body-first versus brain-first model, although some symptoms may be convergent across pathways. Ranges are adapted from the Movement Disorder Society prodromal research criteria and related review literature [4-5,7-9,13-45,61,63]. GABA, gamma-aminobutyric acid

Prodromal symptom	Phenotype	Estimated onset (years before motor onset)	Prevalence (%)	Likelihood ratio	Implicated brain regions	Key neurotransmitters
Rapid eye movement sleep behavior disorder	Body first	5-15	30-60	130-200	Brainstem (pontine tegmentum, locus coeruleus, medullary reticular formation)	GABA, glycine, norepinephrine
Olfactory dysfunction	Brain first	10-15	70-90	4-6	Olfactory bulb, anterior olfactory nucleus, piriform cortex	Dopamine, acetylcholine
Depression	Brain first/convergent	5-7	30-50	1.5-2	Locus coeruleus, dorsal raphe nucleus, prefrontal cortex	Serotonin, norepinephrine
Anxiety	Brain first	4-6	25-40	1.5-2	Amygdala, locus coeruleus	Norepinephrine, serotonin
Apathy	Convergent	2-4	15-25	1.5-2.5	Anterior cingulate cortex, orbitofrontal cortex, basal ganglia	Dopamine, serotonin
Fatigue	Convergent	2-4	20-40	1.3-2	Hypothalamus, brainstem, basal ganglia	Dopamine, serotonin, norepinephrine
Psychosis-related symptoms	Convergent	1-3	10-20	1.2-2	Temporal lobe, limbic cortex, visual association areas	Dopamine, serotonin, acetylcholine
Mild cognitive impairment	Convergent	2-5	20-30	3-4	Prefrontal cortex, hippocampus	Acetylcholine, dopamine

Limitations and research gaps

This review is narrative and non-systematic; accordingly, it is subject to selection bias and limited reproducibility. The evidence base is also heterogeneous, with variability in cohort definitions, ascertainment methods, follow-up duration, and biomarker availability [64-78]. In addition, many neuropsychiatric symptoms are common in the general population and lack specificity for PD when considered alone.

Important gaps remain. Biomarker validation for the earliest metabolic, inflammatory, and synuclein-related changes is still incomplete, while neuroimaging is limited by cost, accessibility, and imperfect specificity [67-76]. More standardized longitudinal studies are needed to determine how neuropsychiatric symptoms evolve and how they interact with biofluid, imaging, genetic, and digital biomarkers for true prodromal risk [77,78].

Clinical implications and future directions

Prodromal neuropsychiatric manifestations may provide a clinically meaningful window for earlier recognition of PD, especially when assessed in a structured combination with other risk markers [9,13,79-82]. Screening instruments such as the REM Sleep Behavior Disorder Screening Questionnaire (RBDSQ) [83], the Geriatric Depression Scale-15 (GDS-15) [84], and the Montgomery-Åsberg Depression Rating Scale (MADRS) [85] may support risk stratification, particularly in patients with idiopathic RBD, hyposmia, autonomic symptoms, or genetic susceptibility [81-82].

Future research should emphasize multimodal prodromal assessment. Digital speech analysis, wearable sensors, passive monitoring, natural language processing, and fluid-based biomarkers may help detect early cognitive and behavioral change non-invasively [67,69,86-87].

## Conclusions

Prodromal neuropsychiatric symptoms are among the earliest clinically relevant manifestations of PD. Interpreting them within a body-first versus brain-first framework helps explain phenotypic heterogeneity and may improve earlier recognition of patients at increased risk. Integrating symptom clusters with imaging, fluid, and digital biomarkers will be essential for moving PD care from late reactive management toward earlier, mechanism-informed intervention.

## Appendices

DECLARATIONS

Acknowledgments

Not applicable.

Funding

The authors received no extramural funding for the study.

Competing interests

The authors declare that they have no competing interests.
